# Programmable state switching based on higher-order exceptional points in anti-parity-time symmetric microcavity systems

**DOI:** 10.1038/s41598-025-13797-4

**Published:** 2025-09-25

**Authors:** Arnab Laha, Dinesh Beniwal, Somnath Ghosh, Adam Miranowicz

**Affiliations:** 1 Institute of Spintronics and Quantum Information, Faculty of Physics and Astronomy, Adam Mickiewicz University, 61-614 Poznan, Poland; 2 School of Cancer & Pharmaceutical Sciences, King’s College London, London, SE1 1UL UK; 3 Department of Physics, École Centrale School of Engineering, Mahindra University, Hyderabad, 500043 India

**Keywords:** Applied optics, Optoelectronic devices and components, Optics and photonics, Microresonators

## Abstract

Diverging from traditional parity-time (PT)-symmetric paradigms, anti-PT (APT) symmetry provides an intriguing framework for harnessing non-Hermitian physics, offering the immense potential to control light-matter interactions in artificial photonic systems reliant on negative-index materials, typically realized with metamaterials. We report a specially configured Fabry-Pérot-type microcavity system by harnessing the unique anti-PT-symmetric constraints with negative-indexed background materials and meticulously balanced gain-loss distributions. We unveil the intriguing topological properties of a parametrically encircled third-order EP (EP3), emerging from two connected second-order EPs (EP2s) among three cavity states. We present a programmable adiabatic state-switching process and highlight the nuanced behaviors of second and third-order branch points by winding around embedded EPs within a 2D gain-loss parameter space. This work explores the theoretical foundations of the topological properties of EPs in negative-indexed media, paving the way for a novel class of metamaterial-based artificial photonic devices.

## Introduction

The integration of non-Hermitian physics into photonic systems has garnered significant interest, particularly within the realm of wave propagation in gain-loss media^1–7^. Non-Hermitian systems typically exhibit complex eigenvalues. However, the distinctive aspect of a particular class of non-Hermitian systems showing parity-time (PT)-symmetry lies in their possession of real eigenvalues in a specific phase^7–11^. A fascinating non-Hermitian phenomenon, the emergence of an exceptional point (EP) singularity, occurs in PT-symmetric systems during the phase transition from an unbroken PT phase (with real eigenvalues) to a broken PT phase (with complex eigenvalues) as control parameters are tuned. An EP of the order *n* (say, EP*n*) is encountered as a topological singularity in the system’s parameter plane when *n* number of underlying eigenvalues and their corresponding eigenvectors coalesce simultaneously^12^. The diverse scientific and technological influence of EPs at the forefront of ongoing research in the fields of photonics facilitate a versatile range of intriguing applications^2–7^, such as controlled lasing with asymmetric state-switching^13–19^, topological state-flipping^20–23^ antilasing^24,25^, slow-light engineering^26^, enhanced nonreciprocity^27–29^ and ultrasensitive detection^30–32^. Moreover, in quantum optics, the intriguing properties of quantum EPs^33–38^ have extensively been studied in the context of quantum state^39^ and process^40^ tomography, quantum heat engines^41,42^, exceptional refrigeration^43^, and many other applications of cutting-edge quantum state engineering. Beyond photonics and quantum optics, the fascinating properties of EPs have also been investigated in atomic^44,45^, molecular^46^, microwave^47^ as well as electronic systems^48^.

Beyond the conventional link between PT-symmetry and EPs, anti-PT (APT) symmetry^49–51^ has recently garnered significant attention in the study of photonic systems utilizing artificial materials. This emerging paradigm presents new opportunities to harness non-Hermitian properties. In an APT-symmetric photonic system with gain and loss, the defining characteristic is the anti-commutation relation between the PT operator and the system’s Hamiltonian. This necessitates a precisely balanced gain-loss distribution embedded within a carefully engineered background composed of both positive and negative refractive index materials (a detailed mathematical framework of an APT-symmetric non-Hermitian Hamiltonian is provided in the next section)^49^. This structural requirement makes APT symmetry different from conventional PT-symmetric systems, where a negative-index background is not essential. The realization of such negative-indexed materials is typically achieved through metamaterials, characterized by negative permittivity and/or negative permeability. Therefore, the integration of EPs within an APT-symmetric system holds substantial promise to unveil novel perspectives in understanding the EP-induced light dynamics^52–55^, particularly influenced by the distinctive attributes of negative-indexed materials.

A recent surge of interest in exploring EP-induced phenomena in APT-symmetric systems has primarily focused on various coupled waveguide configurations with complex coupling^53–55^. However, these studies have mainly addressed second-order EPs (EP2s). The topological nature of EP2s, stemming from their branch point structure in complex parameter spaces, has attracted significant attention, particularly due to the robust, path-independent eigenvalue permutation phenomena that arise during their parametric encirclement, enabling controlled state switching processes. This phenomenon has been widely explored both theoretically^44,45,56^ and experimentally^47,57–60^. Furthermore, state exchange phenomena driven by the parametric encirclement of higher-order EPs have been investigated in waveguide^13,61^ and microcavity systems^21^. However, research on the topological properties of higher-order EPs in APT-symmetric systems, particularly in the context of parametric encirclement, remains lacking. Most of the previous studies have primarily focused on PT-symmetric systems at the transition between exact and broken symmetry regimes. In contrast, systems incorporating negative-index materials, such as metamaterials, lack PT-symmetry and instead exhibit APT-symmetry. A growing interest in this field can be observed in a recent study on APT-symmetric coupled ring resonators for lasing applications^62^. In this article, we leverage the unique characteristics of APT-symmetry to investigate the operation of a Fabry-Pérot-type microcavity system, focusing on its potential for programmable mode-switching applications.

As compered to EP2s in two-level photonic systems, the extended platform to host higher-order EPs in multilevel systems present greater challenges, requiring enhanced complexity in the parameter space with multiple tunable parameters^63^. It has been predicted that the coalescence of *n* coupled states requires $(n^2+n-2)/2$ control parameters^64^, which showcases the complexity of the system’s parameter space in terms of number of control parameters. An alternative approach has been developed^65–67^ based on a concurrent influence of $(n-1)$ EP2s to achieve the topological branch-point behavior inherent to an EP*n*, which has numerically been implemented in waveguide^13^ and microcavity^21^ systems. This alternative approach offers the advantage of reducing the number of required control parameters, simplifying the experimental or numerical handling of the system.

In this paper, we report a specially configured gain-loss assisted APT-symmetric optical microcavity to host parametrically encircled EPs up to order three. Diverging from the conventional paradigm of PT-symmetry, our optimization aims to present the topological features of higher-order EPs under the APT-symmetric constraints using the simplest possible platform. We exclusively design a specialty Fabry-Pérot-type microcavity system incorporating negative-index background materials and a precisely tuned gain-loss profile. Leveraging the interaction of three coupled cavity states in the proximity of two connected EP2s, our proposed system facilitates the exploration of the intriguing topological properties of a third-order EP (EP3). Through appropriate customization of the gain-loss parameter space to accommodate various EP encirclement schemes under APT-symmetric constraints, we reveal the chiral characteristics of both second- and third-order branch points, particularly within the framework of a programmable adiabatic state-switching process. The intertwining aspects of APT-symmetry and exceptional points not only deepen the understanding of fundamental non-Hermitian physics, but also unlock possibilities for designing novel devices with tailored functionalities, spanning the fields of photonics and quantum optics.

## Formation of higher-order EPs in an APT-symmetric system: analytical insights

While, a PT-symmetric Hamiltonian (say, $H_{\text{PT}}$) adheres to the commutation relation $[\text{PT}, H_{\text{PT}}] = 0$ [given that $\text{PT}:\{x,t,i\}\rightarrow \{-x,-t,-i\}$], APT-symmetric Hamiltonian (say, $H_{\text{APT}}$) endorses an anti-commutation relation, i.e., $\{\text{PT}, H_{\text{APT}}\}=0$. Here, $H_{\text{PT}}$ and $H_{\text{APT}}$ adheres an inherent relation $H_{\text{APT}}=\pm iH_{\text{PT}}$. An analytical interpretation of the occurrence of an EP3 in an APT-symmetric system can be perceived by analyzing a $3\times 3$ non-Hermitian Hamiltonian. We consider a general matrix form of a Hamiltonian *H* and a parity operator P as

1 $$\begin{aligned} H=\left( \begin{array}{ccc}a_1-ib_1 & i\alpha & -i\beta \\ i\alpha & a_2-ib_2 & i\alpha \\ -i\beta & i\alpha & a_3-ib_3\end{array}\right) \qquad \text {and}\qquad \text {P}=\left( \begin{array}{ccc}0 & 0 & 1 \\ 0 & 1 & 0 \\ 1 & 0 & 0\end{array}\right) , \end{aligned}$$

where *H* would be APT-symmetric with respect to P under the conditions:

2 $$\begin{aligned} a_3=-a_1,\quad a_2=0, \quad \text{ and }\quad b_1=b_3. \end{aligned}$$

In *H*, the diagonal terms ($a_j-ib_j$ with $j=1,2,3$) represent three unperturbed eigenvalues, where the off-diagonal terms characterized by $\alpha$ and $\beta$ represent complex coupling.

The eigenvalues of *H*, say $\lambda _j\,(j=1,2,3)$, are determined by solving the roots of the cubic equation:

3 $$\begin{aligned} \varepsilon ^3+c_1\varepsilon ^2+c_2\varepsilon +c_3=0. \end{aligned}$$

Under the APT-symmetric conditions given by Eq. (2), the coefficients of Eq. (3) can be expressed as

4a $$\begin{aligned} c_1&= i(2b_1+b_2),\end{aligned}$$

4b $$\begin{aligned} c_2&=-(a_1^2+b_1^2)-2(b_1b_2-\alpha ^2)+\beta ^2 \end{aligned}$$

4c $$\begin{aligned} c_3&= -i\left\{ (a_1^2+b_1^2)b_2-2\alpha ^2(b_1-\beta )-\beta ^2b_2\right\} . \end{aligned}$$

The eigenvalues can be determined using Cardano’s method ^68^, yielding the following expressions:

5a $$\begin{aligned} \varepsilon _1&=\omega \varepsilon _++\bar{\omega }\varepsilon _--\xi , \end{aligned}$$

5b $$\begin{aligned} \varepsilon _2&=\varepsilon _++\varepsilon _--\xi , \end{aligned}$$

5c $$\begin{aligned} \varepsilon _3&=\bar{\omega }\varepsilon _++\omega \varepsilon _--\xi . \end{aligned}$$

Here, $\omega ^3 = 1$, featuring $\omega$ as a cube root of unity, and $\bar{\omega }$ is the complex conjugate of $\omega$. $\varepsilon _{\pm }$ and $\xi$ are given by

6 $$\begin{aligned} \varepsilon _{\pm }=\left( g\pm \sqrt{g^2+h^3}\right) ^{1/3}\quad \text{ and }\quad \xi =\dfrac{c_1}{3}, \end{aligned}$$

where

7 $$\begin{aligned} g=-\dfrac{c_1^2}{27}+\dfrac{c_1c_2}{6}-\dfrac{c_3}{6}\quad \text{ and }\quad h=-\dfrac{c_1^2}{9}+\dfrac{c_2}{3}. \end{aligned}$$

Now, under different settings of the overall perturbation, controlling the interaction among eigenvalues leads to various scenarios involving the emergence of multiple EP2s (pairwise) or an EP3. These scenarios can be understood based on the following conditions:

8 $$\begin{aligned} \varepsilon _+ = \varepsilon _-, \quad \omega \varepsilon _+ = \varepsilon _-, \quad \text{ and } \quad \bar{\omega } \varepsilon _+ = \varepsilon _-. \end{aligned}$$

Each of the conditions in Eq. (8) corresponds to the coalescence of a distinct pair of eigenvalues, resulting in three possible EP2s. Specifically, the condition $\varepsilon _+ = \varepsilon _-$ implies $\varepsilon _1 = \varepsilon _3$, while $\varepsilon _2$ remains distinct. This indicates the presence of an EP2 between the eigenvalue pair $\{\varepsilon _1, \varepsilon _3\}$. Similarly, $\omega \varepsilon _+ = \varepsilon _-$ leads to $\varepsilon _1 = \varepsilon _2 \ne \varepsilon _3$, corresponding to an EP2 between $\{\varepsilon _1, \varepsilon _2\}$. Likewise, $\bar{\omega } \varepsilon _+ = \varepsilon _-$ yields $\varepsilon _2 = \varepsilon _3 \ne \varepsilon _1$, signifying an EP2 between $\{\varepsilon _2, \varepsilon _3\}$.

Together, these conditions indicate that the system can support three distinct pairwise EP2s depending on how the perturbation parameters are tuned. For complete coalescence of all three eigenvalues, i.e., the occurrence of an EP3 (where $\varepsilon _1 = \varepsilon _2 = \varepsilon _3$), all three conditions in Eq. (8) must simultaneously hold. This is achieved under the more restrictive requirement that $\varepsilon _+ = \varepsilon _- = 0$, corresponding to the vanishing of both cube roots in Cardano’s formalism. However, here we focus on exploring the topological branch-point behavior of an EP3 by winding around any of the two connected EP2s, where the fulfillment of any two of the three conditions in Eq. (8) is mandatory.

Moreover, the validation of the equalities in Eq. (8) indicates that the square root term in $\varepsilon _{\pm }$ [given by Eq. (6)] vanishes, leading to the cube-root dependence of $\varepsilon _{\pm }$ directly. It is important to highlight that when we focus on a single EP2, the analytical problem simplifies to a $2\times 2$ Hamiltonian, where two associated eigenvalues feature square-root terms^56^. Hence, the sensitivity of such a system follows a square-root dependence on perturbation at an individual EP2. But when we consider a $3\times 3$ Hamiltonian, the eigenvalues consists cube root terms, and hence the sensitivity exhibits a cube-root dependence on perturbation, like the case of an EP3.

Now, we implement such a coupling scheme in an APT-symmetric photonic system, where the complex potential is represented by the complex refractive index profile *n*(*x*). Here the adherence to APT-symmetry is contingent upon the condition $n(x)=-n^*(-x)$ [unlike a PT-symmetric system with $n(x)=n^*(-x)$]. This requirement results in the real part of the refractive index behaving as an odd function, while the imaginary part exhibits characteristics of an even function; i.e., $n_{\text{R}}(x)=-n_{\text{R}}(-x)$ and $n_{\text{I}}(x)=n_{\text{I}}(-x)$ with $n(x)=n_{\text{R}}(x)+in_{\text{I}}(x)$. In this context, the variable $n_{\text{I}}$ is fundamentally tied to the interplay of gain and loss within an optical system. Therefore, the implementation of APT-symmetry demands a balanced and symmetrical distribution of gain and loss across a structured background index profile (like a PT-symmetric system). However, the odd function characteristics of $n_{\text{R}}$ introduce an additional condition for an APT-symmetric system, mandating the use of negative-indexed background materials alongside a precisely balanced gain-loss distribution^49^. This fundamental engineering difference distinguishes an APT-symmetric system from PT-symmetric systems.

Therefore, we implement such a complex coupling scheme, as mathematically demonstrated by the Hamiltonian in Eq. (1), in a gain-loss assisted multiplayer microcavity system with a combination of positive and negative refractive indexed materials. A carefully engineered layer-by-layer gain–loss modulation, based on a chosen set of parameters, introduces imaginary components into the effective permittivity, which is manifested as complex-valued couplings in the corresponding Hamiltonian. Moreover, the asymmetry in the coupling strengths (i.e., distinct values of $\alpha$ and $\beta$, as in the Hamiltonian) can be precisely tuned by adjusting the thickness and refractive index contrast of the individual layers, as well as by independently detuning the real and imaginary parts of the refractive index across the structure. Therefore, the interplay among index contrast, gain-loss modulation, and layer thickness governs the complex mode coupling in the system, effectively giving rise to the off-diagonal complex terms in the effective non-Hermitian Hamiltonian.

To analyze our microcavity system, we implement scattering (*S*) matrix formalism method, where the physical eigenvalues can be calculated in terms of the poles of the associated *S*-matrix. Here, an EP3 emerges from the interplay between two interconnected EP2s. Instead of directly encountering an EP3, which requires a complex coupling structure with many variables, we focus on its topological properties. This is achieved by encircling two interconnected EP2s within a 2D parameter space based on gain-loss profile, as delineated in the following sections.

**Fig. 1:**
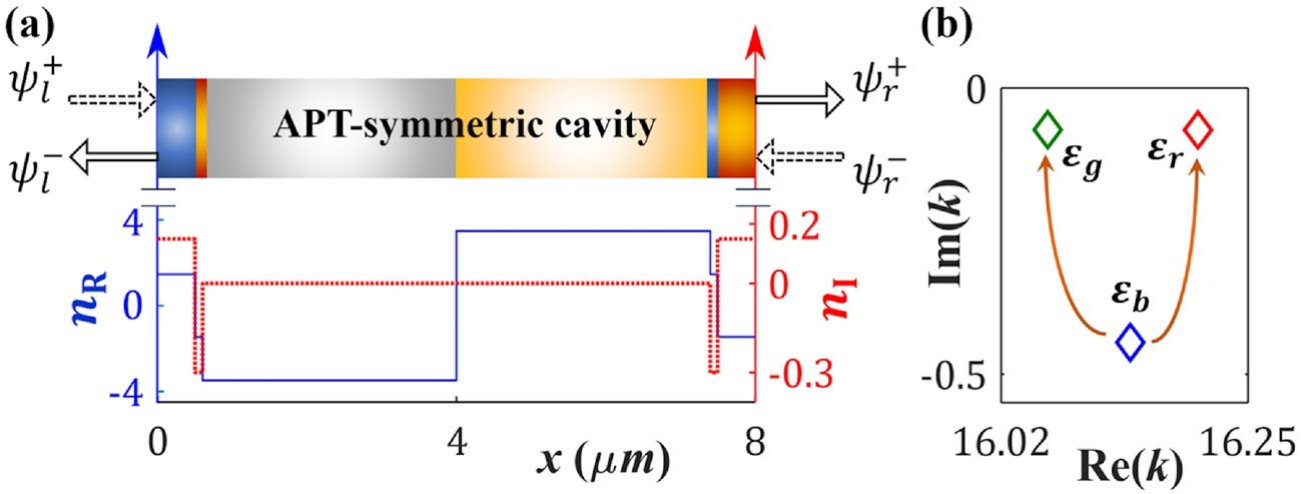
(**a**) A schematic representation of the proposed APT-symmetric microcavity consists of six layers [labeled as L1 to L6 from left to right, as mentioned in Eq. (9)], along with the corresponding complex refractive index profile *n*(*x*). In the schematic, $\psi _{{l(r)}}^{+}$ and $\psi _{{l(r)}}^{-}$ represent the forward and backward propagating fields, respectively, from the left (right) side of the cavity. In the refractive index profile, the variations of the $n_{\text{R}}(x)$ and $n_{\text{I}}(x)$ are illustrated by solid blue and dotted red lines, respectively (labeled along the left and right *y*-axes). (**b**) The coordinates of three chosen *S*-matrix poles, indicated as $\varepsilon _r$, $\varepsilon _b$, and $\varepsilon _g$, situated within the complex *k*-plane, while considering the passive cavity with $\gamma =0$. Two arrows show the anticipated interaction scheme among them in the proximity of two connected EP2s.

## Results and discussion

### Designing an APT-symmetric microcavity

We engineer a 1D Fabry-Pérot type optical microcavity with a blend of positive and negative indexed [say, $n_{\text{R}}(x)$] background materials, featuring a length of $L=8\,\mu m$ $(0\le x\le L)$. Here, non-Hermiticity is achieved through a tailored gain-loss profile, typically represented by the imaginary part of the refractive index [say, $n_{\text{I}}(x)$], where negative $n_{\text{I}}(x)$ corresponds to gain and positive $n_{\text{I}}(x)$ corresponds to loss. In this configuration, the overall cavity system consists of six layers [designated as L1 to L6 from left to right side, as in Eq. (9)] distinguished by two passive index parameters, $\pm n_1=3.48$ and $\pm n_2=1.46$, along with two gain-loss control parameters, $\gamma$ (a gain-loss coefficient) and $\tau$ (a loss-to-gain ratio). The overall refractive index profile *n*(*x*), expressed as $n(x)=n_{\text{R}}(x)+in_{\text{I}}(x)$, operates according to the functional form:

9 $$\begin{aligned} n(x)=\left\{ \begin{array}{l} \pm \,n_1\\ \pm \,n_2-i\gamma \\ \mp \,n_2+i\tau \gamma \end{array}\right. \hspace{-0.25cm} \begin{array}{l} :|x-l_0|\in [0,\,l_1]\rightarrow \text{ L4 } \text{ and } \text{ L3 },\\ :|x-l_0|\in [l_1,\,l_2]\rightarrow \text{ L5 } \text{ and } \text{ L2 },\\ :|x-l_0|\in [l_2,\,l_3]\rightarrow \text{ L6 } \text{ and } \text{ L1 }. \end{array} \end{aligned}$$

The entire setup is designed to maintain APT-symmetry, which can be understood by the distributions of $n_{\text{R}}(x)$ and $n_{\text{I}}(x)$ based on precisely chosen length parameters $l_j$ $(j=0,1,2,3;\,l_j<L)$ with $l_0=4\,\mu m=l_3$, $l_1=3.4\,\mu m$, and $l_2=3.5\,\mu m$. Figure 1a shows a schematic design of the entire cavity system, accompanied by the chosen profile of complex *n*(*x*), where the corresponding $n_{\text{R}}(x)$ and $n_{\text{I}}(x)$ follow the features of odd and even functions, respectively. The chosen configuration allows us to uphold APT-symmetry consistently for any specified values of $\gamma$ and $\tau$ throughout our investigation. Figure 1b illustrates the positions of three complex eigenvalues, which are analyzed to explore their interactions in the presence of gain-loss. These eigenvalues are determined based on the poles of the *S*-matrix associated with the microcavity, as explained in the following methods section.

With a universal approach, our optimization aims to simplify the system’s geometry, making it easier for practical realization. We choose the Fabry-Pérot geometry due to its straightforward practical design and broad material availability. Moreover, the length of a Fabry-Pérot-type cavity has precise control on the spacing between corresponding resonance states over a particular frequency range. Such a configuration offers seamless integration into existing optical systems and allows for axial output coupling, which is infeasible in toroidal resonators without proper phase matching. Our proposed design offers an ease of scalability to different sizes (including miniaturization) and frequencies. Notably, there are no active components in the two inner layers that span the majority of our designed cavity ($0.6\,\mu m\le x\le 7.4\,\mu m$ from the total cavity-length of $8\,\mu m$). Instead, the gain-loss profile confined to the four thin outer layers exclusively controls the entire coupling and subsequent interactions within the cavity. Such a configuration provides a convenient platform for practical implementations.

Using state-of-the-art fabrication techniques, a similar scalable prototype can be achieved with silica-silicon-based materials for the positive-indexed layers, while optical metamaterials can be manipulated^69^ to achieve the desired negative refractive index for the negative-indexed layers under the operating conditions. The customized gain-loss profile can be integrated via a controlled nonuniform pumping scheme or by doping of lossy and gain elements using a standard lithography technique. In this context, the effect of material dispersion can be controlled by careful engineering of both the material properties and the microcavity geometry based on chosen frequency range. Due to precise control of the Fabry-Pérot-type cavity geometry on the spacing between resonance states, the proposed multilayered structure, incorporating custom-engineered materials and metamaterials, offers enhanced control over material dispersion while operating within the selected narrow frequency range (from 15.5 $\mu m^{-1}$ to 16.5 $\mu m^{-1}$; even after considering the movement of poles under proposed encirclement conditions, as exhibited later).

### Method: scattering matrix formulation

**Fig. 2:**
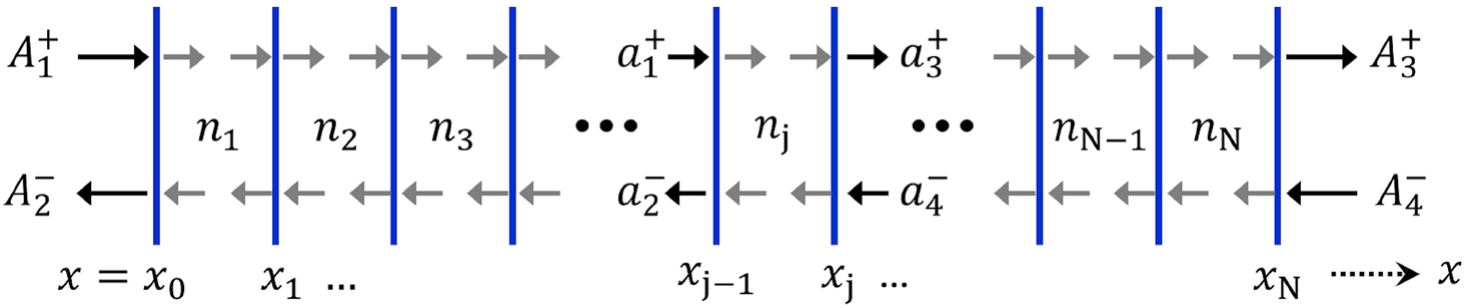
Schematic diagram of a 1D two-port open multilayer microcavity system composed of *N* layers with refractive indices $n_1, n_2, \ldots , n_{\text {N}}$. The field amplitudes at the extreme left and right ends of the system are denoted by $\{A^+_1,\, A^-_2\}$ and $\{A^+_3,\, A^-_4\}$, respectively. The modified field amplitudes at positions $x=x_{{j-1}}$ and $x=x_{{j}}$, corresponding to the *j*th layer, are given by $\{a^+_1,\, a^-_2\}$ and $\{a^+_3,\, a^-_4\}$.

Here, we manifest the physical eigenvalues in terms of the resonance states of the designed microcavity, which are numerically estimated by the scattering (*S*) matrix formalism^21,70^. The *S*-matrix relates these incoming and outgoing field amplitudes from the both sides. For a 1D multilayer microcavity system, the *S*-matrix can be derived by combining the transfer matrices of individual layers (based on the scattering theory of electromagnetism). Figure 2 schematically represents a 1D multilayer microcavity system composed of *N* layers, which is structurally analogous to our proposed system. The field amplitudes at the extreme left and right ends of the system are denoted by $\{A^+_1,\, A^-_2\}$ and $\{A^+_3,\, A^-_4\}$.

Each of the layers can be described by a transfer matrix *T*, which relates the field amplitudes at the right side of the layer to those at the left side. Considering a specific *j*th-layer that occupies the region $x=x_{{j-1}}$ to $x=x_{{j}}$, the transfer matrix equation can be written as

10 $$\begin{aligned} \left[ \begin{array}{l} a^+_3 \\ a^-_4 \end{array}\right] _{x\,=\,x_{{j}}}= T_j \left[ \begin{array}{l} a^+_1 \\ a^-_2 \end{array}\right] _{x\,=\,x_{{j-1}}}, \end{aligned}$$

where, $\{a^+_1,\,a^-_2\}$ and $\{a^+_3,\,a^-_4\}$ are the modified field amplitudes at left ($x=x_{{j-1}}$) and right ($x=x_{{j}}$) sides of the layer (as shown in Fig. 2). $T_j$ represents the $2\times 2$ transfer matrix of the *j*th-layer. The overall transfer matrix *T* of the multilayer system is the product of the transfer matrices of the individual layers:

11 $$\begin{aligned} T=T_1.T_2.T_3\ldots T_j\ldots T_N, \end{aligned}$$

where *N* represents the total number of layers. Here, the order of multiplication corresponds to the physical sequence of layers in the cavity. The matrix elements of the overall transfer matrix

12 $$\begin{aligned} T=\left[ \begin{array}{ll} T_{11} & T_{12} \\ T_{21} & T_{22} \end{array}\right] \end{aligned}$$

are the functions of frequency and chosen refractive index.

Now, the *S*-matrix that relates incoming and outgoing field amplitudes [as described by Eq. (15)] can be written as

13 $$\begin{aligned} S\left\{ n(x),k\right\} =\left[ \begin{array}{ll} S_{11} & S_{12} \\ S_{21} & S_{22} \end{array}\right] =\left[ \begin{array}{ll} R^{{(l)}} & T^{{(r)}} \\ T^{{(l)}} & R^{{(r)}} \end{array}\right] . \end{aligned}$$

Here, *R* and *T* represent the reflection and transmission coefficients from left (*l*) and right (*r*) side of the cavity, respectively. The *S*-matrix elements can be computed in terms of the *T*-matrix elements as follows:

14 $$\begin{aligned}&S_{11}=R^{{(l)}}=-\dfrac{T_{21}}{T_{22}},\qquad&S_{12}=T^{{(r)}}=\dfrac{1}{T_{22}},\nonumber \\&S_{21}=T^{{(1)}}=\dfrac{\text{ det }(T)}{T_{22}},\qquad&S_{22}=R^{{(r)}}=\dfrac{T_{12}}{T_{22}}. \end{aligned}$$

Therefore, the *S*-matrix equation for the overall microcavity system can be written as

15 $$\begin{aligned} \left[ \begin{array}{l} A^-_2 \\ A^+_3 \end{array}\right] = S\left\{ n(x),k\right\} \left[ \begin{array}{l} A^+_1 \\ A^-_4 \end{array}\right] . \end{aligned}$$

We implement this process numerically to analyze our cavity. Equation (15) essentially represents the *S*-matrix equation for our proposed microcavity, where the field amplitudes $\{A^+_1,\,A^-_4\}$ and $\{A^-_2,\,A^+_3\}$ are associated with incoming fields $\{\psi _{{l}}^{+},\,\psi _{{r}}^{-}\}$ and outgoing fields $\{\psi _{{l}}^{-},\,\psi _{{r}}^{+}\}$.

The matrix elements $S_{ij}\{k,n(x)\}$ are derived as functions of both frequency (*k*) and the chosen *n*(*x*). Adhering to the energy conservation and causality conditions, the complex poles of the *S*-matrix, residing in the fourth quadrant of the complex *k*-plane with $\text{Re}(k)=m\pi/(n_{\text{R}}L)$ (where *m* denotes the order of the poles; $m=1,2,3\ldots$), signify the physical resonance states within the cavity^21,70^. These poles are determined by solving the equation

16 $$\begin{aligned} \dfrac{1}{\max \left| \text{eig}\left[ S\left\{ n(x),k\right\} \right] \right| }=0 \end{aligned}$$

through a numerical root-finding method. From a large number of poles appearing in the lower half of the complex *k*-plane, we meticulously choose a set of three poles within the frequency range $16.02\le \text{Re}(k) \le 16.25$ (in $\mu m^{-1}$) to study our interaction schemes. These three chosen poles are denoted as $\varepsilon _r$, $\varepsilon _b$, and $\varepsilon _g$, where their distribution in the complex *k*-plane are shown in Fig. 1b (indicated by three diamond markers of red, blue, and green colors, respectively). It is noteworthy that we can also choose other frequency range to observe similar interaction phenomena. The initial distribution of these poles in the complex *k*-plane follows a nonlinear pattern, primarily resulting from the chosen nonuniform profile of $n_{\text{R}}(x)$. With the onset of non-Hermiticity through controlled adjustments of gain-loss parameters, $\gamma$ and $\tau$, these poles become mutually coupled. Such a coupling in response to changes in the control parameters stems from variations in the resonant frequencies (energies) and decay rates (lifetimes) [as indicated by Re(*k*) and Im(*k*), respectively] of the corresponding poles. We delve into their interactions, exhibiting avoided-crossing characteristics in the proximity of two second-order branch points.

### Hosting EPs of different orders

**Fig. 3:**
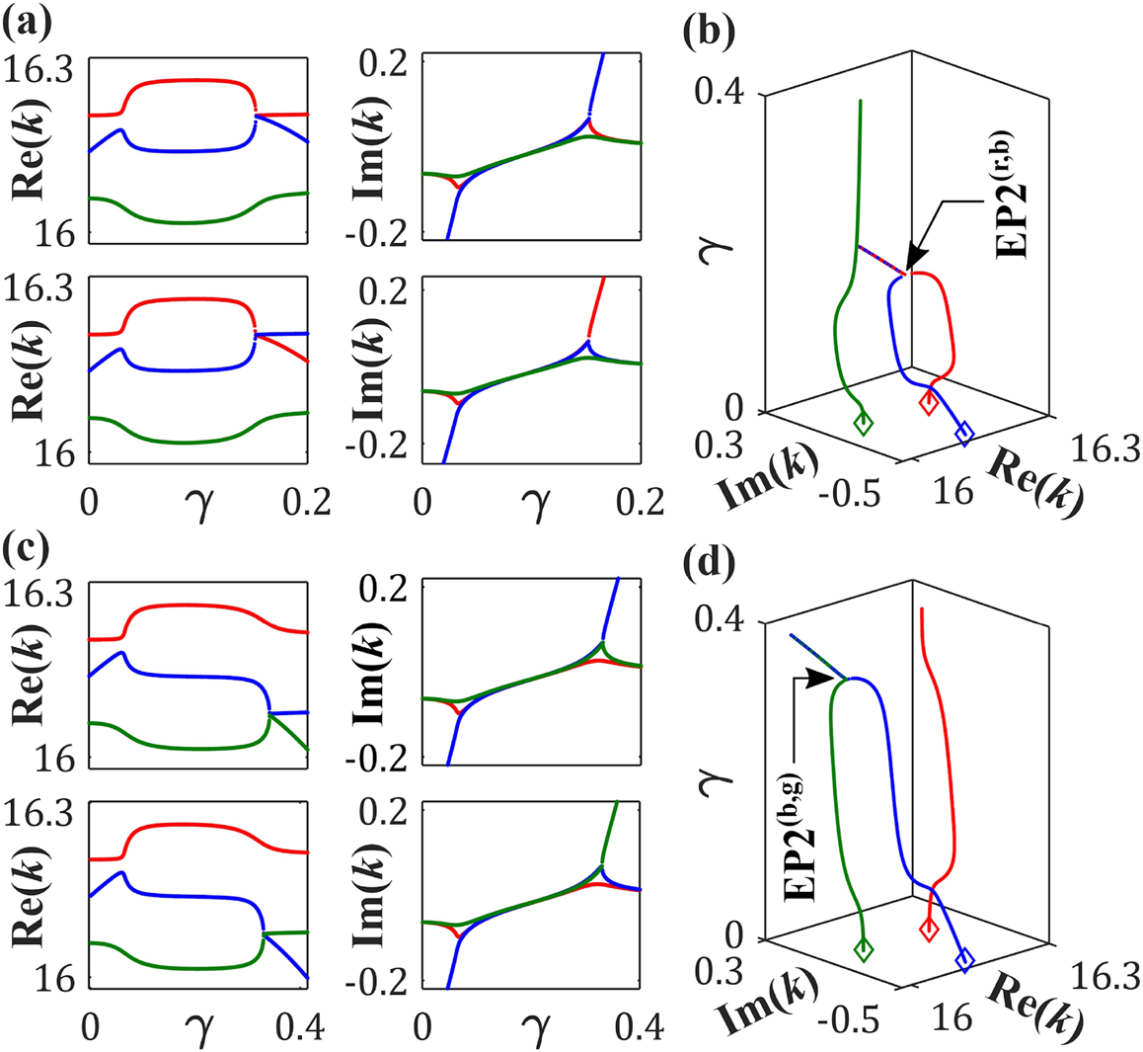
Trajectories of $\varepsilon _r$, $\varepsilon _b$, and $\varepsilon _g$ (depicted by dotted red, blue, and green curves) with an increasing $\gamma$, while considering different $\tau$-values. (**a**) $\tau =0.879$ (upper panel): An anticrossing and a crossing in Re(*k*) and Im(*k*), respectively, associated with $\varepsilon _r$ and $\varepsilon _b$, occurring near $\gamma =0.1525$. $\tau =0.883$ (lower panel): A crossing and an anticrossing in Re(*k*) and Im(*k*), respectively, associated with $\varepsilon _r$ and $\varepsilon _b$, occurring near $\gamma =0.1535$. (**b**) $\tau =0.881$: Emergence of EP2$^{\text{(r,b)}}$ due to the coalescence of $\varepsilon _r$ and $\varepsilon _b$ at $\gamma =0.153$. $\varepsilon _g$ remains away from the strong interaction regime of $\varepsilon _r$ and $\varepsilon _b$ in (**a**) and (**b**). (**c**) $\tau =0.275$ (upper panel): An anticrossing and a crossing in Re(*k*) and Im(*k*), respectively, associated with $\varepsilon _b$ and $\varepsilon _g$, occurring near $\gamma =0.3305$. $\tau =0.279$ (lower panel): A crossing and an anticrossing in Re(*k*) and Im(*k*), respectively, associated with $\varepsilon _b$ and $\varepsilon _g$, occurring near $\gamma =0.3315$. (**d**) $\tau =0.277$: Emergence of EP2$^{\text{(b,g)}}$ due to the coalescence of $\varepsilon _b$ and $\varepsilon _g$ at $\gamma =0.331$. $\varepsilon _r$ remains away from the strong interaction regime of $\varepsilon _b$ and $\varepsilon _g$ in (**c**) and (**d**). In (**b**) and (**d**), the diamond markers show the locations of $\varepsilon _r$, $\varepsilon _b$, and $\varepsilon _g$ at $\gamma =0$. The unit of *k* is $\mu m^{-1}$.

We monitor the trajectories of $\varepsilon _r$, $\varepsilon _b$, and $\varepsilon _g$ in Fig. 3, while deviating from the passive condition through a gradual increase of $\gamma$, across various $\tau$-values. In Fig. 3a, we depict two different topological configurations of avoided-crossings among $\varepsilon _r$ and $\varepsilon _b$ in the complex *k*-plane, where $\varepsilon _g$ deviates from the interaction regime. While considering $\tau =0.879$, Re(*k*) associated with $\varepsilon _r$ and $\varepsilon _b$ undergo an anticrossing, and the corresponding Im(*k*)-values exhibit a crossing with an increasing $\gamma$ (as shown in the upper panel). However, a slight increase in $\tau$ to 0.883 unfolds an exactly opposite topological scenario (for the same variation of $\gamma$) with a crossing and an anticrossing in Re(*k*) and Im(*k*), respectively, linked with $\varepsilon _r$ and $\varepsilon _b$ (as shown in the lower panel). Such a topological transition utterly validates the occurrence of a singularity, specifically an EP2. Here, we identify an EP2, say EP2$^{\text{(r,b)}}$, for an intermediary $\tau =0.881$, leading to coalescence of $\varepsilon _r$ and $\varepsilon _b$ at $\gamma \approx 0.153$. In this case, $\varepsilon _g$ remains unaffected. In a similar way, two topologically different avoided-crossings among $\varepsilon _b$ and $\varepsilon _g$ with an anticrossing (a crossing) in Re(*k*) and a crossing (an anticrossing) in Im(*k*) can be observed in the upper panel (lower panel) of Fig. 3c, while varying $\gamma$ for a chosen $\tau =0.275$ ($\tau =0.279$). This implies the emergence of another EP2, say EP2$^{\text{(b,g)}}$, as shown in Fig. 3d, where $\varepsilon _b$ and $\varepsilon _g$ coalesce at $\gamma \approx 0.331$ for an intermediary $\tau =0.277$, keeping $\varepsilon _r$ unaffected.

Therefore, we observe a unique scenario involving the three chosen poles, where $\varepsilon _b$ becomes analytically connected to $\varepsilon _r$ and $\varepsilon _g$ through two interconnected EP2s, i.e., EP2$^{\text{(r,b)}}$ and EP2$^{\text{(b,g)}}$, positioned at coordinates (0.153, 0.881) and (0.331, 0.277), respectively, within the $(\gamma ,\tau )$-plane (2D parameter space). Notably, while a specific pair of poles coalesce at an EP2, the third pole remains unaffected. Such an intricate scenario occurring within a particular interaction regime leads to the emergence of a third-order branch point, specifically an EP3, where all three interacting poles are intricately linked^21,65^. The significance of our study lies in adopting a universal methodology that remains valid even when considering APT-symmetry. Our distinctive cavity configuration ensures the persistence of APT symmetry over the entire adjustable range of $\gamma$ and $\tau$. While the complex poles, typically in the broken PT phase, may enter an unbroken PT phase for specific values of $\gamma$ and $\tau$, this PT-phase transition is not associated with the occurrence of an EP. Without relying on any inherent connection between EPs and PT-phase transitions, our proposed approach is effective for identifying multiple connected EP2s, facilitating the exploration of the topological properties of higher-order EPs.

### Parametrically encircling the encountered EPs

We investigate the branch-point behaviors of the embedded EP2s by driving the perturbation quasistatically in terms of a patterned gain-loss variation along a closed loop in the $(\gamma ,\tau )$-plane, subject to various conditions under the APT-symmetric constraints. When such a parametric loop encloses one or more EP2s, the associated eigenvalues undergo a cyclic permutation that remains invariant with respect to the specific shape or size of the loop, as long as the enclosed EP2s remain within the loop. Consequently, neither any deformations nor fluctuations in the loop trajectory affect the eigenvalue permutation. This robust behavior reflects the branch-point topology of the eigenvalue planes and is thus referred to as topological in nature. It is worth noting, although not the focus of this manuscript, that encircling an EP can also induce a geometric phase^57^ (specifically, a Pancharatnam–Berry phase), even in the absence of dynamic evolution. This phase is linked to the holonomy (or nontrivial loop structure) in parameter space, which further reinforces the topological character of EP encirclement phenomena.

For our analysis, we consider a particular loop defined parametrically as

17 $$\begin{aligned} \gamma (\phi )=\gamma _0\sin (\phi /2)\quad \text{ and }\quad \tau (\phi )=\tau _0-p\sin \phi . \end{aligned}$$

Here, $(\gamma _0,\tau _0)$ and $p,\,(<1;\,\ne 0)$ represent the characteristic parameters determining the number of EP2s to be encircled ($\gamma _0$ must exceed the $\gamma$-coordinate of the respective EP2 to be encircled), where $p>0\,(p<0)$ characterizes an anticlockwise (a clockwise) encirclement scheme for $0\le \phi \le 2\pi$. The chosen shape of the parametric loop ensures that the encirclement process begins ($\phi =0$) and ends ($\phi =2\pi$) at the passive cavity condition ($\gamma =0$).

**Fig. 4:**
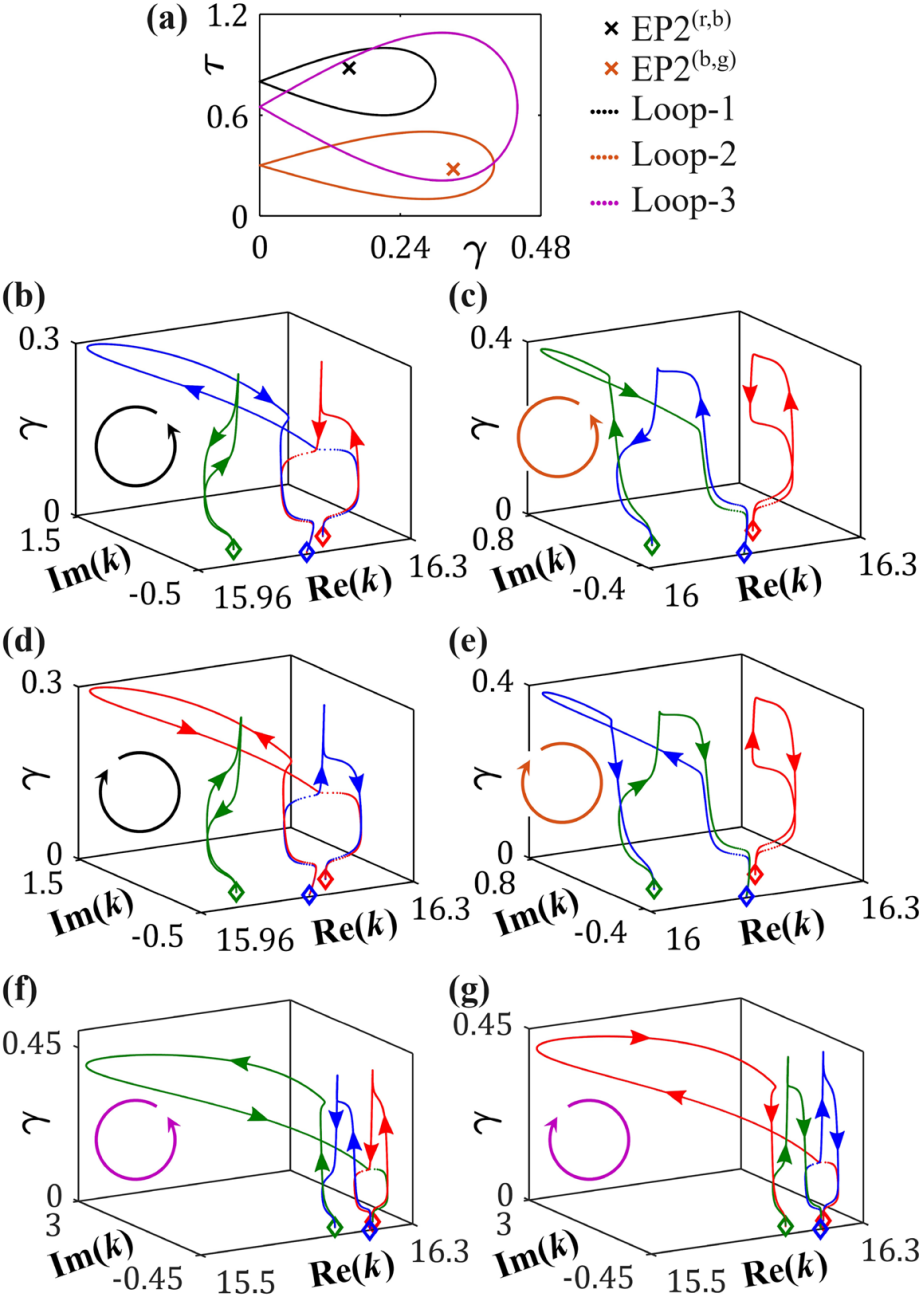
(**a**) The coordinates of EP2$^{\text{(r,b)}}$ and EP2$^{\text{(b,g)}}$, along with three chosen quasistatic encirclement schemes. Loop-1 and Loop-2 individually encircle EP2$^{\text{(r,b)}}$ and EP2$^{\text{(b,g)}}$, respectively, whereas Loop-3 encircles both EP2s simultaneously. (**b**–**g**) Trajectories of $\varepsilon _r$, $\varepsilon _b$ and $\varepsilon _g$ (depicted by dotted red, blue, and green curves, respectively) in the complex *k*-plane (*k* in $\mu m^{-1}$), while considering the variations of $\gamma$ and $\tau$ under various conditions: (**b**) along Loop-1 in the anticlockwise direction, exhibiting the adiabatic permutations $\varepsilon _r\rightarrow \varepsilon _b\rightarrow \varepsilon _r$ and $\varepsilon _g\rightarrow \varepsilon _g$; (**c**) along Loop-2 in the anticlockwise direction, exhibiting the adiabatic permutations $\varepsilon _b\rightarrow \varepsilon _g\rightarrow \varepsilon _b$ and $\varepsilon _r\rightarrow \varepsilon _r$; (**d**) along Loop-1 and (**e**) Loop-2 in the clockwise direction, where alteration of trajectories between the exchanging poles and reversed movement of the third pole compared to (**b**) and (**c**) are evident; (**f**) along Loop-3 in the anticlockwise direction, exhibiting the adiabatic permutation $\varepsilon _r\rightarrow \varepsilon _b\rightarrow \varepsilon _g\rightarrow \varepsilon _r$; (**g**) along Loop-3 in the clockwise direction, exhibiting the adiabatic permutation $\varepsilon _r\rightarrow \varepsilon _g\rightarrow \varepsilon _b\rightarrow \varepsilon _r$. In (**b**–**g**), the diamond markers show the locations of $\varepsilon _r$, $\varepsilon _b$, and $\varepsilon _g$ at $\phi =0$.

It is important to note that although the gain–loss profile is externally modulated in accordance with the parametric loop, it is intrinsically tied to the underlying geometry (structure and complex refractive indices) of the system. The spatial distribution of gain and loss, realized through carefully engineered multilayer configurations, acts as the physical substrate through which the parametric variation is implemented. Thus, the control parameters ($\gamma$ and $\tau$) trace out the loop by virtue of geometric and material design, ensuring a direct connection between the abstract parametric space and the actual physical structure. Moreover, while varying $\gamma$ and $\tau$ along a closed loop, the Kramers-Kronig relations play a crucial role, as these parameters are linked to $n_{\text{I}}(x)$ over the chosen $n_{\text{R}}(x)$. It is essential to maintain precise control over $n_{\text{I}}(x)$ to ensure that slight modifications in $n_{\text{R}}(x)$ do not cause the EP to shift outside the loop. In our proposed microcavity, the absence of gain-loss in majority of the structural geometry ($0.6\,\mu m\le x\le 7.4\,\mu m$), combined with the robustness of EP-induced topological properties against reasonable parametric tolerances^20^, ensures the persistence of the Kramers-Kronig relations for our study.

Here, the features of the second-order branch point of the embedded EP2s become evident when encircling them individually. However, an encirclement scheme enclosing both the EP2s simultaneously reveals the nature of a third-order branch point. Figure 4a illustrates the coordinates of EP2$^{\text{(r,b)}}$ and EP2$^{\text{(b,g)}}$, alongside three distinct encirclement schemes in the $(\gamma ,\tau )$-plane. The encirclement patterns are delineated as follows: Loop-1 (the black loop), characterized by $\gamma _0=0.3$, $\tau _0=0.8$, and $p=0.2$, encloses only EP2$^{\text{(r,b)}}$; Loop-2 (the orange loop), governed by $\gamma _0=0.4$, $\tau _0=0.3$, and $p=0.2$, encloses EP2$^{\text{(b,g)}}$ solely; whereas Loop-3 (the violet loop), characterized by $\gamma _0=0.44$, $\tau _0=0.65$, and $p=0.44$, encircles both the connected EP2s, simultaneously. In this context, we note that the gain-loss variation along a chosen parametric loop does not significantly influence nearby poles (other than three interacting poles associated with the two connected EP2s), provided no additional EP2 involving any of the selected poles is enclosed by the loop. Moreover, from a practical standpoint, minimizing the loop size is preferable, since a larger loop demands a higher gain-loss modulation amplitude, which may pose challenges for implementation due to increased pumping requirements. Therefore, a parametric loop must be carefully optimized to balance functional requirements with experimental feasibility.

The topological effects induced by the chosen encirclement schemes are investigated by tracing the trajectories of $\varepsilon _r$, $\varepsilon _b$, and $\varepsilon _g$, as portrayed in Figs. 4b–4g. Here, each point of evolution on the trajectory of a specific pole in the complex *k*-plane aligns with a corresponding point of evolution on a specific loop in the $(\gamma ,\tau )$-plane. In this context, the poles may undergo either unbroken or broken PT phases as the parameters $\gamma$ and $\tau$ are varied, in accordance with Eq. (17).

Now, while considering an anticlockwise encirclement by varying $\gamma$ and $\tau$ quasistatically along Loop-1 [that encircles only EP2$^{\text{(r,b)}}$, and keeps EP2$^{\text{(b,g)}}$ outside], the poles $\varepsilon _r$ and $\varepsilon _b$, which are connected through EP2$^{\text{(r,b)}}$, exchange their initial positions adiabatically in the complex *k*-plane. Upon completing a full $2\pi$ rotation along the loop, $\varepsilon _r$ and $\varepsilon _b$ completely swap their frequencies, transitioning as $\varepsilon _r\rightarrow \varepsilon _b\rightarrow \varepsilon _r$, as depicted in Fig. 4b. Nevertheless, this structured perturbation around EP2$^{\text{(r,b)}}$ does not impact $\varepsilon _g$ [i.e., $\varepsilon _g\rightarrow \varepsilon _g$, as can be observed in Fig. 4b, which remains at the same frequency level at the end of the encirclement process. In a similar fashion, a complete $2\pi$ anticlockwise parametric rotation along Loop-2 [that encircles only EP2$^{\text{(b,g)}}$, keeping EP2$^{\text{(r,b)}}$ outside] results in an adiabatic frequency-swapping between $\varepsilon _b$ and $\varepsilon _g$ (like, $\varepsilon _b\rightarrow \varepsilon _g\rightarrow \varepsilon _b$), while leaving $\varepsilon _r$ unaffected (i.e., $\varepsilon _r\rightarrow \varepsilon _r$), as illustrated in Fig. 4c. The unconventional interactions, as observed among the three cavity states in Figs. 4b and 4c, showcasing distinct state-flipping characteristics within two corresponding pairs, unfold the individual second-order branch-point behavior of EP2$^{\text{(r,b)}}$ and EP2$^{\text{(b,g)}}$. In this context, a $2\pi$ clockwise rotation of $\gamma$ and $\tau$ along both Loop-1 and Loop-2 results in a similar permutation among the cavity-states, as shown in Figs. 4d and 4e. It is notable that two exchanging poles alters their trajectories, where the third one moves along the opposite directions [as compared to the trajectories for anticlockwise encirclement schemes, as previously shown in Figs. 4b and 4c. Therefore, the trajectories of the poles (Figs. 4b–4e) under parametric encirclement around individual EP2s, in both clockwise and anticlockwise directions, convey the individual chiral property of both the embedded EP2s. Here, to restore the initial frequencies by the chosen cavity states, a complete $4\pi$ rotation is required for the encirclement schemes along Loop-1 and Loop-2 in any of the directions.

**Table 1:** Programmable state-switching process induced by gain-loss distribution described by Loop-3 (in the proximity of an EP3 associated with two interconnected EP2s). Here, $n\circlearrowleft$ and $n\circlearrowright$ mean $2n\pi$ anticlockwise and clockwise rotations, respectively.

Initial states	Final states		
	$\varepsilon _r$	$\varepsilon _b$	$\varepsilon _g$
$\varepsilon _r$	3 $\circlearrowleft$ or 3 $\circlearrowright$	1 $\circlearrowleft$ or 2 $\circlearrowright$	2 $\circlearrowleft$ or 1 $\circlearrowright$
$\varepsilon _b$	2 $\circlearrowleft$ or 1 $\circlearrowright$	3 $\circlearrowleft$ or 3 $\circlearrowright$	1 $\circlearrowleft$ or 2 $\circlearrowright$
$\varepsilon _g$	1 $\circlearrowleft$ or 2 $\circlearrowright$	2 $\circlearrowleft$ or 1 $\circlearrowright$	3 $\circlearrowleft$ or 3 $\circlearrowright$

To delve into the intriguing properties of an EP3 as a third-order branch point, we consider a quasistatic variation of $\gamma$ and $\tau$ along Loop-3, encompassing both EP2$^{\text{(r,b)}}$ and EP2$^{\text{(b,g)}}$ simultaneously. Such a patterned perturbation interestingly facilitates a topological switching among all three interacting poles interconnected via EP2$^{\text{(r,b)}}$ and EP2$^{\text{(b,g)}}$. Notably, a complete $2\pi$ rotation in the anticlockwise direction results in a successive and adiabatic exchange of frequencies among $\varepsilon _r$, $\varepsilon _b$ and $\varepsilon _g$, following the sequence $\varepsilon _r\rightarrow \varepsilon _b\rightarrow \varepsilon _g\rightarrow \varepsilon _r$ within the complex *k*-plane, as shown in Fig. 4f. This manifestation vividly showcases the third-order branch point behavior of an EP3 in the presence of interconnected EP2s. Furthermore, the effect of a complete $2\pi$ rotation in the clockwise direction along Loop-3 can be distinguished from the sequence of the resulting successive state exchange phenomena, as illustrated in Fig. 4g. Here, we can observe a successive and adiabatic frequency switching phenomenon such as $\varepsilon _r\rightarrow \varepsilon _g\rightarrow \varepsilon _b\rightarrow \varepsilon _r$, unlike the case for the anticlockwise encirclement process. This disparity underscores a breakdown of the chiral property alongside the presence of a third-order branch point, i.e., an EP3. Such a breakdown of chirality offers a promising avenue for implementing a programmable state-switching mechanism in the proximity of an EP3 (i.e., along the violet loop enclosing both the connected EP2s), as depicted in Table 1. This table outlines the required rotations, either clockwise or anticlockwise, for the transition between states. Notably, a full $6\pi$ rotation (in any of the directions) is necessary to revert to the initial cavity states.

## Conclusions

In conclusion, this research delves into the intricate characteristics of higher-order EPs within a specialty gain-loss assisted optical microcavity adhering to APT-symmetry. Beyond the widely explored connection between EPs and PT-symmetry, the inclusion of APT-symmetry adds a new dimension to the physics dealing with the topological interplay of gain-loss and negative refractive indexed synthetic materials and expands the repertoire of platforms available for manipulating light. We specifically focus on exploring the topological properties of an EP3 associated with two connected EP2s among three cavity states. We investigate various state-exchange mechanisms driven by the topological characteristics of these second- and third-order branch points, while examining different encirclement schemes in the gain-loss parameter space. A successive and adiabatic switching process is revealed among up to three cavity states. It is important to note that our chosen APT-symmetric microcavity configuration allows for the encounter of EPs without being tied to PT-phase transitions. Furthermore, leveraging the intriguing chiral aspects uncovered, we explore a programmable state-switching scheme as a potential application of the designed APT-symmetric microcavity. These findings contribute significantly to our comprehension of integrating non-Hermitian physics into classical wave-based systems reliant on metamaterials, thereby advancing the development of artificial devices for all-photonic applications. Implementation of our proposed scheme has significant importance in achieving multi-state chiral dynamics for device applications, where the central idea can be translated into other scalable metamaterial-based multicore fiber or multimode planar waveguide structures with gain and loss, enabling unconventional light guidance schemes with mode conversion and one-way transmission.

## Data Availability

Data sets generated during the current study are available from the corresponding author on reasonable request.
